# Trajectories of illness perceptions in patients with kidney disease receiving dialysis: Relationship with psychological and physical symptoms

**DOI:** 10.1371/journal.pone.0323814

**Published:** 2025-05-15

**Authors:** Buse Keskindag, Ken Farrington, Duriye Deren Oygar, Sam Norton, Shivani Sharma

**Affiliations:** 1 School of Life and Medical Sciences, University of Hertfordshire, Hatfield, United Kingdom; 2 Psychology Department, Bahçeşehir Cyprus University, Nicosia, Northern Cyprus, Turkey; 3 Burhan Nalbantoglu State Hospital, Nicosia, Northern Cyprus, Turkey; 4 Institute of Psychiatry, Psychology and Neuroscience, King’s College London, London, United Kingdom; 5 College of Business and Social Sciences, Aston University, Birmingham, United Kingdom; MSH Medical School Hamburg, GERMANY

## Abstract

Illness perceptions play an important role in outcomes for patients with advanced kidney failure receiving dialysis, though they are rarely examined over a sustained period and in distinct cultural settings. This observational cohort study used a prospective longitudinal design to examine how illness perceptions change overtime. It also considered whether these trajectories are related to patient experience of psychological and physical symptoms including those associated with dialysis, and depressive mood. Data were collected from 181 patients recruited from four dialysis centres in Northern Cyprus between 2020–2021. There were 124 patients receiving dialysis (91.1% haemodialysis) and 57 patients in the pre-dialysis phase at baseline. Self-reported measures including the Brief Illness Perception Questionnaire, Patient Health Questionnaire-9, and Dialysis Symptom Index, were completed at the start (time 1) of the study and then again at six months (Time 2) and at 12 months (Time 3) using versions validated in the local language (Turkish). Multilevel Models (MLM) for repeated measures were used to understand trajectories of illness perceptions over the 12-months of follow-up. On average, perceptions of consequences and emotional response to illness decreased over a one-year period. Depressive symptoms and dialysis symptom burden were found to be relatively stable over the same period. However, patients who reported higher perceptions of illness consequences and emotional response at baseline were more likely to report greater depressive symptoms at 12 months. Similarly, those already receiving dialysis who reported greater emotional response and lower levels of personal control at baseline were more likely to continue to report higher dialysis symptom burden at 12 months. The findings underscore the importance of illness perceptions as a framework to identify patients who may benefit from support, importantly offering an anchor for intervention design. Establishing cultural acceptability of such an approach will be an important next step.

## Introduction

Chronic kidney disease (CKD) affects 9% of the population across the globe [1]. Its prevalence has increased over the last 20 years [2], mainly attributed to higher rates of diabetes and hypertension [3]. Data suggest that burden is variable across countries, with low and middle income settings seeing higher demand for public health prevention, and specialist intervention [1,4]. CKD is classified into 5 stages, with the most advanced stage (stage 5) necessitating kidney replacement therapy (KRT). Intervention for earlier stages of CKD is aimed at slowing down progression and preventing complications [5]. Individuals with advanced kidney failure (CKD stage 5) have higher levels of disease burden, higher levels of hospitalization and greater risk for mortality [2].

Worldwide, kidney failure is most commonly treated by dialysis, of which there are two main modalities- haemodialysis (HD) and peritoneal dialysis (PD) [6]. Longitudinal assessments underscore the way in which psychological factors such as depression complicate outcomes for dialysis patients with those with clinically significant symptoms being at higher risk for hospitalization, dialysis withdrawal and mortality [7,8]. Patients receiving dialysis have high symptom burden attributed to the physical and lifestyle demand of this modality as compared to a kidney transplant [9]. Symptom burden associated with dialysis such as fatigue, low blood pressure, itching/ pruritus, poor sleep as well as experience of depressive mood is more common as compared to individuals with CKD not receiving KRT [10–12]. Recent review studies show that patients with CKD, including those who are not receiving KRT, experience similar profile of symptoms such as fatigue, general pain, poor mobility [13,14]. Symptom burden across spectrum of CKD with or without KRT makes management critical.

The common-sense model (CSM) of self-regulation of health and illness [15] offers a framework to better understand various behavioural and emotional responses to long term conditions. The CSM explains that individuals develop cognitive and emotional representations regarding a health threat to make sense of their condition. These representations inform the coping strategies which can contribute to illness outcomes such as mental and physical functioning [16]. Illness perceptions represent a patient’s beliefs about illness timeline (chronicity of illness and cyclicality of illness course), causes, controllability (both by treatment and personal behaviours), the somatic symptoms they attribute to their illness (identity) and the overall impact of illness (consequences) [17]. Illness perceptions have been shown to influence emotional and behavioural responses [15,17]. Using the illness perception questionnaire, research in general has shown associations between mental well-being and perceptions of illness consequences, control/cure, and timeline among patients with different medical conditions [18]. Favourable illness perceptions (higher levels perceptions of coherence and control) have been found to relate to better health outcomes, whilst unfavourable illness perceptions (higher perceptions of consequences, emotional representation, and timeline) related to poorer outcomes [19].

As CKD encompasses patients with a broad range of kidney function, it is likely that illness experiences will vary between individual’s dependants on CKD stage. Further, for the individuals, illness experience may change overtime. Trajectories of illness perceptions have not been studied extensively. The research that does exists suggests that illness perceptions of patients in the pre-dialysis phase versus those receiving dialysis vary due to treatment burden [20]. Patients receiving dialysis tend to perceive greater illness consequences than those in the pre-dialysis phase [20]. Perceptions of treatment control and timeline have also been shown to increase when patients commence dialysis [21]. Though patients in the pre-dialysis phase have different treatment other than dialysis such as medication, negative illness perceptions (e.g., greater illness consequence and emotional response) were associated with poorer physical and mental health in this patient group [22]. However, these perceptions may change over time.

In a six-year prospective study, patients receiving HD reported stronger perceptions of illness chronicity and lower illness consequences at follow-up [23]. Similarly, patients receiving HD indicated better illness coherence and lesser emotional response to their illness after two-years of dialysis [24]. These findings indicate a more optimistic view at follow-up. Overall, prospective studies suggest that identifying negative illness perceptions early on may facilitate implementation of interventions to bolster illness outcomes [25].

The majority of research on illness perceptions in CKD has originated from high income countries in the west. This makes it difficult to understand how far this framework can support patient experience and outcomes across cultural settings, though the illness perceptions framework itself has been widely applied across cultural contexts. Keskindag et al. [26] have previously shown in Turkish Cypriot patients receiving HD that illness perceptions appear to be an acceptable framing to support patients to express their experience of living with CKD. Limited other research has examined illness perceptions of patients receiving dialysis in Turkey [27,28]. This limits the extent to which local health services can draw on findings to drive attempts to support patients.

The primary aim of this study was to understand whether illness perceptions change over time among patients with advanced CKD – both pre-dialysis and in patients established on dialysis (PD or HD). The research also examined trajectories of depressive symptom and dialysis symptoms. Based on past evidence [20–22,24,29–36], the research addressed the following objectives in Turkish Cypriot people with CKD- 1) whether illness perceptions change over 12 months period for those pre-dialysis or currently dialysing it 2) how depressive symptoms change over time, 3) whether different levels of illness perceptions relate to change in depressive symptoms, 4) how dialysis symptoms change over time, 5) whether different levels of illness perceptions relating to change in dialysis symptoms.

## Materials and methods

### Study design and setting

Ethical permission was obtained from the Lefkosa Dr Burhan Nalbantoglu State Hospital, Department of Inpatient Treatment, Ministry of Health in Turkish Republic of Northern Cyprus (YTIKI1.01-629-20/E.1915). Potential participants were approached through four renal services in four different state hospitals in Northern Cyprus. These hospitals were the only health centres that provide renal services in Northern Cyprus at that time. A quantitative prospective longitudinal design was employed to understand patient’s illness perceptions at three different time points. The participants in the pre-dialysis phase (expected to start dialysis within 6 months [Time 1]) were followed up at the commencement of dialysis [within the first month = Time 2] and at 6 months after receiving dialysis [Time 3)]). Patients already receiving dialysis completed the same questionnaire pack when they were first approached [Time 1]), at 6 months (Time 2) and after 1 year (Time 3). The Strengthening the Reporting of Observational Studies in Epidemiology (STROBE) checklist was used to report this study (S1 Table).

### Participants

All patients receiving dialysis and those who were expected to start dialysis within 6 months were invited to participate. The inclusion criteria were: 1) patient expected to start dialysis treatment within 6 months 2) those already receiving dialysis treatment (HD or PD), 3) verbal fluency in Turkish, 4) aged over 18 years. Exclusion criteria were 1) mental health issues (referred or treated within the previous year) 2) lack of capacity to consent 3) receiving end of life care. Data collection for both groups were completed between 1^st^ July 2020 and 30^th^ October 2021. Data collection was initially face to face and shifted to via telephone during the course of the research owing to COVID-19 restrictions. With a prevalent dialysis population of 211 patients, an initiation rate of 58 patients/year and assuming an acceptance rate to participate of 60%, we expected to recruit 160 patients.

### Data collection

Once the participants agreed to join the study, they were asked to provide written informed consent. At this stage they completed a questionnaire pack measuring variables including demographic attributes; age, gender, marital status and employment. For each measurement occasion (i.e., Time 1, Time 2 and Time 3) they also completed the following questionnaires, Brief Illness Perception Questionnaire (Brief IPQ) [37], Patient Health Questionnaire-9 (PHQ-9) [38], and Dialysis Symptom Index (DSI) [39]. With explicit consent, medical data from routine blood reports (i.e., creatinine, albumin, and haemoglobin, dialysis adequacy [Kt/V] values) were collected during planned visits to the hospitals at each time point also. Co-occurring conditions were recorded with help of renal nurses at the relevant centres.

### Measurements

All questionnaires were implemented in the local native language (Turkish). 

#### Brief Illness Perception Questionnaire (Brief IPQ).

The Brief IPQ includes nine items assessing the views of the patients related to their illness across domains of the CSM of self-regulation of health and illness [15]. The benefit of the Brief IPQ is that a single item is used to assess each dimension of the illness perceptions framework, making the scale time efficient to complete without compromising measurement [37]. It uses a 0 -to- 10 response scale. Items assess cognitive illness representations, emotional representations, causal beliefs, and illness comprehensibility. High score in each item refers to increases in the component measured. For instance, high score on perception of consequences refers to greater perception related to impact of the illness [37]. Concurrent, predictive and discriminant validity has been demonstrated along with test re-test reliability [37]. Oflaz and colleagues translated the questionnaire into Turkish [40]. The translated version shows good level content validity [41].

**Patient Health Questionnaire-9 (PHQ-9).** The PHQ-9 was designed to measure depressive symptoms over the previous two weeks. It includes 9 items and they are assessed by using 4-point (0–3) scale. PHQ-9 is a reliable and valid measure of depressive symptom severity [36]. Scores of 5–9, 10–14, 15–19, and over 20 refer to mild, moderate, moderately severe, and severe depressive symptom, respectively. Güleç and colleagues translated the scale into Turkish and demonstrated criterion and construct validity [42]. In the current study, items of PHQ-9 scale indicated a good internal reliability score of 0.81.

**Dialysis Symptom Index (DSI).** The DSI was developed to assess symptom prevalence and severity of dialysis during the previous 7 days. It consists of 30 items evaluated using 5-point response scale (0–4). High scores refer to greater symptom burden. Content validity and test-retest reliability has been demonstrated [39]. Onsoz and Usta Yesilbalkan translated the index into Turkish, demonstrating good content validity and internal consistency [43]. In the current study, items of DSI demonstrated high internal reliability score of 0.89.

### Data analysis

Descriptive statistics and longitudinal data analyses were conducted in Jamovi version 1.6.23 [44]. Socio-demographic data patterns were explored in descriptive terms such as the characteristics of the patients in terms of age, gender, marital status, employment, clinical values, and comorbidity status (number of comorbid conditions). Visual exploration of the data showed that the dependent variables (*perceptions of consequences, timeline, emotional response, personal control, depressive symptoms,* and *dialysis symptoms* (six variables in total) seemed to be normally distributed. Longitudinal data analysis was conducted using Multilevel Models (MLM) for repeated measures deploying the maximum likelihood estimation technique to examine growth within and between individuals and their predictors [45,46]. For each dependent variable (*perceptions of consequences, timeline, emotional response, personal control, depressive symptoms,* and *dialysis symptoms* (six variables in total), a separate MLM analysis was conducted. Time was entered as a continuous variable as months from baseline assessment. Assessments of whether predictor variables were associated with increasing or decreasing level of the outcome were assessed by using cross-product interaction terms between predictor and time. It was ensured that multicollinearity was not present. Normality of residuals was tested and examined via Q-Q plot. Homogeneity of variance of residuals was visually examined by scatterplot. Additionally, chi-square analysis and t-tests were performed to examine attrition in the study by considering demographic factors and illness perceptions of those who did not complete data collection at all three time points.

## Results

### Patient recruitment and retention

**Fig 1:**
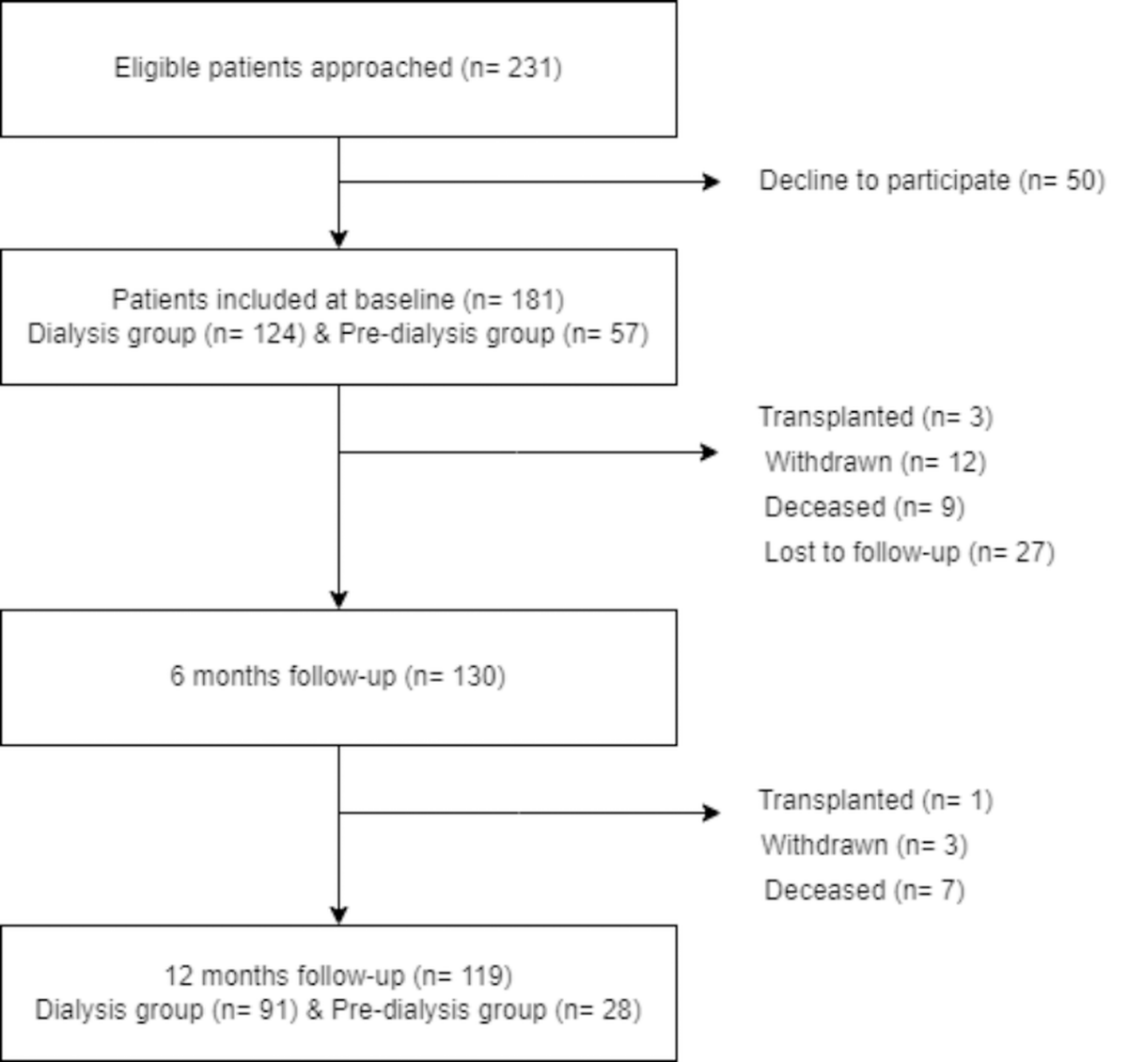
Patient recruitment and engagement with study.

In total 231 patients were approached for the study. Baseline patient recruitment included 124 patients who were already receiving dialysis and 57 patients who were within the pre-dialysis phase and expected to commence dialysis within 6 months (Fig 1). Some participants were not assessed at the Time 2 and Time 3 due death (n = 16), transplanted (n = 4) and self-withdrawal from the study (n = 15). Data collection at Time 1 was completed face to face, however, data collection at Time 2 and Time 3 were completed by telephone because restrictions associated with COVID-19. Only 10 patients moved from pre-dialysis to dialysis over the course of the study (at 12 months follow-up). This low number was likely to be due to restricted access to healthcare and patient reluctance to attend during the pandemic. Such factors had a negative impact on follow-up particularly of this subgroup, hence, we did not perform the full analyses for these patients.

### Baseline patient characteristics

Table 1 presents demographic, clinical, and psychosocial characteristics at baseline for both groups (i.e., dialysis and pre-dialysis). The majority of patients were male, married, and retired. Mean age was > 60 years in both groups. Among reported diagnoses, uncertain aetiology (44.1%) was the most common diagnosis which was followed by diabetic nephropathy (28%). In the dialysis group, the majority of patients received HD treatment in a hospital setting (91.1%).

**Table 1:** Characteristics of patients at baseline.

	Patients on pre-dialysis phase (n = 57)	Patients receiving dialysis (n = 124)
**Demographic**		
Age (mean, SD)	62.8 (13.8)	64.3 (14.9)
Gender (male n, %)	42 (73.2%)	85 (68.5%)
Marital status (n, %)		
Married	45 (78.6%)	90 (72.5%)
Single/divorced/widowed	12 (21.1%)	33 (26.6%)
Employment (n, %)		
Retired	39 (68.4%)	80 (64.5%)
Full time/part time employee	5 (8.7%)	6 (4.8%)
Housewife*	9 (15.7%)	26 (19.3%)
Unemployed	4 (7.1%)	12 (9.6%)
**Clinical**		
Dialysis vintage (in years, mean SD)	–	4.53 (4.34)
Treatment (n, %)		
Haemodialysis	–	113 (91.1%)
Peritoneal dialysis	–	11 (8.9%)
Creatinine (umol/L, mean SD)	302 (115)	771 (215)
Albumin (g/l, mean SD)	4.13 (0.48)	3.93 (0.37)
Haemoglobin (g/L, mean SD)	114 (18.2)	110 (18.6)
Dialysis efficiency (Kt/V, mean SD)	–	1.59 (0.48)
	*Overall (n = 181)*	
Diagnosis (n, %)		
Uncertain aetiology	41 (44.1%)	
Diabetic nephropathy	26 (28%)	
Polycystic kidney	9 (9.7%)	
Hypertension	7 (7.5%)	
Other	10 (10.9%)	
**Psychosocial**		
Brief IPQ (mean, SD)		
Consequences	3.39 (3.36)	7.40 (3.35)
Timeline	6.92 (3.06)	8.10 (2.74)
Personal Control	4.81 (2.30)	2.79 (2.36)
Treatment Control	7.47 (2.57)	8.23 (2.21)
Identity	1.32 (1.99)	5.07 (3.36)
Concern	3.46 (3.69)	3.27 (3.72)
Coherence	7.34 (3.78)	7.22 (3.57)
Emotional response	3.98 (3.44)	5.26 (3.83)
PHQ-9 (mean, SD)	3.63 (3.86)	7.02 (3.86)
DSI (mean, SD)	–	50.8 (22.9)

Brief IPQ, Brief Illness Perception Questionnaire; PHQ-9, Patient Health Questionnaire-9; DSI, Dialysis Symptom Index; *There were only women in this group.

The mean level of depressive symptoms (7.02) in the dialysis group aligns with mild depressive symptoms at baseline in line with established cut off scores [38]. In addition 30.2% of the patients in the dialysis group scored 10 and above on PHQ-9, which may suggest a possible clinically significant condition [38]. This patient group remained in the sample as none were referred or received mental health treatment within the previous year. Similarly, the DSI scores indicate high symptom burden [39].

Analysis of those who dropped out of the study did not reveal any differences in demographic or specific illness perception factors (S2 Table).

### Longitudinal data analyses

#### Change in illness perceptions over time.

Table 2 summarises MLMs for perceptions of consequences, personal control, emotional response, and timeline separately. There were significant variations in perceptions of consequences, emotional response, and timeline at baseline (random intercept), suggesting between-patient variability.

**Table 2:** Multilevel models for illness perceptions over a one year follow up.

	Consequences	Emotional response	Personal Control	Timeline
Fixed effects (Estimate [SE])				
Intercept	5.94 (0.64)***	5.27 (0.69)***	3.48 (0.49)***	6.99 (0.60)***
Time	-0.47 (0.20)*	-0.64 (0.22)**	0.65 (0.17)***	0.69 (0.21)***
Age	-0.02 (0.02)	-0.05 (0.01)***	-0.02 (0.01)*	0.05 (0.01)***
Comorbidity status	-0.01 (0.22)	-0.01 (0.23)	-0.05 (0.15)	0.19 (0.19)
Pre-dialysis – Dialysis	-3.43 (0.83)***	-1.09 (0.92)	-0.03 (0.69)	-0.74 (0.87)
Female – Male	0.48 (0.45)	1.69 (0.47)***	-0.01 (0.31)	0.49 (0.38)
Time x Pre-dialysis – Dialysis	-0.80 (0.41)*	-0.66 (0.46)	2.30 (0.34)***	0.17 (0.43)
Random components				
Within-patients (L1) variance	6.55	8.50	4.69	3.30
Intercept (L2) variance	2.96	2.57	0.63	2.00
Slope (L2) variance	–	–	–	–
Additional information				
ICC	0.311	0.223	0.119	0.377
AIC	1663	1724	1458	1065
BIC	1697	1758	1492	1096
Likelihood Ratio Chi-square Tests				
Random intercept	20.8***	13.8***	3.7	13.5***
Random slope	–	–	–	–
(marginal) Pseudo *R*^2^	.34	.18	.44	.16

*p < 0.05 **p < 0.01***p < 0.001.

For consequences, the slope for time (average effect of time) was -0.47 which was significant, indicating that perception of consequences was expected to decrease 0.47 units at each measurement occasion that passes beyond the first measurement occasion (i.e., 6 months). Considering the expected perception of consequences at baseline was 5.94, the expected perception of consequences in one year would be 5 (β_*average mean of intercepts *_*+ β*_*fixed effect of time variable*_ (2) *=* 5.94 + [-0.47(2)] = 5). Perception of consequences for the dialysis group were expected to be 3.43 units higher than the pre-dialysis group at baseline and the slope of interaction between time and different phases (i.e., pre-dialysis and dialysis) was significant, indicating a different rate of change in the two phases. However, whilst the rate of change in the dialysis group was not significant at -0.07, that for the pre-dialysis group was -0.88, suggesting that perception of consequences was expected to decrease over time.

For emotional response the slope for time was significant at -0.64, suggesting an expected decrease in emotional response at each measurement occasion (i.e., 6 months). The slope of the interaction between time and different phases (pre-dialysis and dialysis) was not significant. However, whilst the rate of change in the dialysis group not significant at -0.31, that in the pre-dialysis group was at -0.97, suggesting an expected decrease in the pre-dialysis group over time.

For personal control the slope for time was significant at 0.65, suggesting an increase in personal control at each measurement occasion (i.e., 6 months). The rate of change in personal control was different in pre-dialysis and dialysis groups. In the dialysis group it was significant at -0.50 suggesting an expected decrease in perception of personal control over time. On the other hand, the rate of change among the pre-dialysis group was at 1.80 and it was significant, suggesting an expected increase in perceptions of personal control over time.

For timeline, the slope for time was significant at 0.69 suggesting an expected increase in the perception of timeline (chronicity) at each measurement occasion (i.e., 6 months). The slope of interaction between time and different phases (i.e., pre-dialysis and dialysis) was not significant. However, the expected rate of change in the dialysis group was significant at 0.61 suggesting an expected increase in perception of timeline in the dialysis group over time. Similarly, the expected rate of change among the pre-dialysis group was significant at 0.78 units suggesting an increase in the perception of timeline in pre-dialysis patients.

**Change in depressive symptoms over time.** Five different MLMs were run to observe changes in depressive symptoms over time (Table 3). In the first model, where depressive symptom scores were the dependent variable, coefficients of time, age, comorbidity status, different phases (i.e., pre-dialysis and dialysis group), gender and the interaction of time and different phases were observed. Variance between patients’ mean depressive symptom scores (intercept) was significant, suggesting that patients vary in terms of depressive symptom scores at baseline. The slope for time was not significant at -0.12. Baseline depressive symptom scores for the dialysis group were expected to be 3.46 units higher than pre-dialysis group. Expected rates of change in the groups were not significant at -0.15 and -0.08 respectively, suggested stability of depressive symptom scores in both groups.

**Table 3:** Multilevel models for depressive symptoms over a one year follow up.

	Depressive symptoms	Depressive symptoms^a^	Depressive symptoms^b^	Depressive symptoms^c^	Depressive symptoms^d^
Fixed effects (Estimate [SE])					
Intercept	4.53 (0.90)***	4.67 (0.82)***	4.17 (0.76)***	5.03 (0.89)***	5.48 (1.06)***
Time	-0.12 (0.25)	-0.003 (0.21)	0.13 (0.21)	-0.15 (0.22)	-0.33 (0.28)
Consequences^a^		0.34 (0.12)**			
Emotional response^b^			0.43 (0.12)***		
Personal Control^c^				-0.07 (0.18)	
Timeline^d^					-0.14 (0.23)
Age	-0.01 (0.02)	0.01 (0.02)	0.02 (0.02)	-0.01 (0.02)	-0.01 (0.02)
Comorbidity status	0.49 (0.32)	0.49 (0.30)	0.47 (0.28)	0.39 (0.32)	0.32 (0.37)
Pre-dialysis – Dialysis	-3.46 (1.06)***	-1.72 (0.67)*	-2.56 (0.57)***	-2.94 (0.71)***	-3.67 (0.87)***
Female – Male	2.32 (0.66)***	2.11 (0.62)***	1.49 (0.57)*	2.45 (0.66)***	2.41 (0.76)**
Time x Pre-dialysis – Dialysis	-0.06 (0.50)				
Time x Consequences^a^		0.04 (0.05)			
Time x Emotional response^b^			0.03 (0.05)		
Time x Personal Control^c^				-0.05 (0.08)	
Time x Timeline^d^					0.14 (0.13)
Random components					
Within-patients (L1) variance	9.57	8.87	8.67	9.71	10.43
Intercept (L2) variance	8.07	6.71	5.11	7.55	8.66
Slope (L2) variance	–	–	–	–	–
Additional information					
ICC	0.458	0.431	0.371	0.437	0.454
AIC	1832	1800	1771	1788	1355
BIC	1866	1837	1809	1826	1390
Likelihood Ratio Chi-square Tests					
Random intercept	50.6***	40.5***	27.0***	42.5***	27.1***
Random slope	–	–	–	–	–
(marginal) Pseudo *R*^2^	.18	.27	.33	.21	.16

a, b, c, d represent separate models including only one illness perception domain as predictor (i.e., consequences, emotional response, personal control and timeline respectively).

*p < 0.05 **p < 0.01***p < 0.001.

Additional MLMs were conducted in which one of the selected illness perception domains (i.e., consequences, emotional response, personal control, and timeline) was entered as an additional predictor. All models showed that there were significant variations between patients in terms of their depressive symptom scores at baseline (Table 3). In the second model, the slope for perception of consequences was significant at 0.34, suggesting that depressive symptom scores were expected to increase by 0.34 unit with each increment of perception of consequences. In the third model, the slope for emotional response was significant at 0.43 indicating an increase in depressive symptom score with each increment in emotional response. In the fourth model and fifth models the slopes for perception of personal control and perception of timeline were not significant suggesting neither predicted change in depressive symptoms.

**Change in dialysis symptom burden over time.** Using data on dialysis group, five models were run to observe change in dialysis symptom burden over one year period (Table 4). In the first model, the rate of change in dialysis symptoms was not significantly different between patients receiving PD and those receiving HD treatment. However, dialysis symptom burden was predicted to increase 3.79 units with each increment in the number of comorbid conditions (Table 4).

**Table 4:** Multilevel models for dialysis symptom burden over a one year follow up.

	Dialysis symptom burden	Dialysis symptom burden^a^	Dialysis symptom burden^b^	Dialysis symptom burden^c^	Dialysis symptom burden^d^
Fixed effects (Estimate [SE])					
Intercept	39.32 (7.60)***	43.19 (4.98) ***	41.12 (4.76)***	49.16 (5.93)***	48.65 (6.39)***
Time	1.06 (2.95)	-0.16 (1.26)	0.61 (1.22)	-1.41 (1.33)	-2.15 (1.55)
Consequences^a^		1.76 (0.92)			
Emotional response^b^			2.17 (0.71)**		
Personal Control^c^				2.62 (1.28)*	
Timeline^d^					-0.01 (1.35)
Age	-0.01 (0.11)	0.06 (0.10)	0.10 (0.09)	-0.01 (0.11)	-0.04 (0.13)
Comorbidity status	3.79 (1.65)*	3.78 (1.47)*	3.82 (1.38)**	3.62 (1.64)*	2.80 (1.73)
PD – HD	-11.32 (13.99)	-2.14 (6.39)	-2.06 (6.08)	4.45 (7.65)	-4.80 (8.42)
Female – Male	6.62 (3.48)	5.17 (3.10)	2.34 (3.00)	8.39 (3.50)*	5.88 (3.63)
Time x PD – HD	2.72 (5.93)				
Time x Consequences^a^		0.32 (0.47)			
Time x Emotional response^b^			0.13 (0.34)		
Time x Personal Control^c^				-2.01 (0.65)**	
Time x Timeline^d^					0.61 (0.81)
Random components					
Within-patients (L1) variance	244	225	217.4	227	262
Intercept (L2) variance	152	103	87.8	152	128
Slope (L2) variance	–	–	–	–	–
Additional information					
ICC	0.384	0.315	0.288	0.401	0.328
AIC	2108	2074	2059	2064	1706
BIC	2140	2109	2094	2099	1738
Likelihood Ratio Chi-square Tests					
Random intercept	22.1***	13.9***	11.9***	23.4***	11.0***
Random slope	–	–	–	–	–
(marginal) Pseudo *R*^2^	0.06	.20	.25	.10	.06

a, b, c, d represent separate models involving only one illness perception domain as predictor (i.e., consequences, emotional response, personal control and timeline respectively).

*p < 0.05 **p < 0.01***p < 0.001.

In the second model, the slope for perception of consequences was not significant, indicating that perception of consequences did not predict change in dialysis symptom burden. In the third model, dialysis symptom burden was expected to increase 2.17 unit with increase in emotional response scores. In the fourth model, dialysis symptom burden was expected to increase 2.62 units with each increment in perception of personal control at baseline. However, the slope for interaction between time and perception of personal control was found -2.01 and was significant, suggesting that perception of personal control significantly influenced rate of change in dialysis symptom burden over time. Further analysis showed that dialysis symptom burden was predicted to decrease 6.12 units at each measurement occasion (i.e., 6 months) among patients who reported a higher level of perception of personal control than average. In the fifth model, perception of timeline did not predict change in dialysis symptom burden.

## Discussion

The primary aim of this study was to observe illness perception trajectories over time among patients with kidney disease, specifically those in the pre-dialysis phase or those already receiving dialysis. Both aspects of the patient journey may be challenging and for different reasons such as patients receiving dialysis experiencing social restrictions due to scheduled dialysis sessions whilst patient in the pre-dialysis phase changing their habits for better health (e.g., dietary changes, medication) [47,48]. Understanding illness representations and their relation to psychological factors may be helpful therefore to planning stage appropriate support.

We have confirmed that illness perceptions change over one year period. Depressive symptoms were found to be relatively stable over the same time frame, but those patients who reported higher perceptions of consequences and emotional response at baseline also had greater depressive symptoms. Similarly, dialysis symptoms were found to be stable over time amongst those receiving intervention. Nevertheless, patients reporting greater emotional response at baseline were more likely to report more severe dialysis symptom burden over time and, those who reported a high level of personal control had less severe symptoms.

The findings align with existing research that shows that illness perceptions are not static, they may change over time [49]. Though there is limited longitudinal data in the context of CKD, the current findings add to this evidence base further echoing the importance of considering how such trajectories relate to broader aspects of patient experience and quality of life [20,22,24]. Overall scores in both pre-dialysis and dialysis patient groups showed that patients’ perceptions of consequences tend to decrease overtime. However, this slightly varied among patient groups, further analysis showed perception of consequences was predicted to decrease significantly in pre-dialysis group at 12 months. Similarly, emotional response tend to decrease in both patient groups over one year period, further analysis indicated perception of emotional response patients in the pre-dialysis phase was predicted to decrease significantly over time. Although overall scores of perceptions of personal control in both groups tend to increase, further analysis illustrated the scores tend to decrease significantly in the patient group receiving dialysis whilst it was predicted to increase significantly in pre-dialysis patient group at 12 months. Additionally, perception of timeline (chronicity) tend to increase in both patient groups showing significant increase in scores of perception of timeline. Understanding illness perceptions in CKD is not straightforward, as findings indicate illness perceptions may vary at different stages.

The reduced perceptions of consequences and emotional response may be related to time on treatment/phase, which may help to normalise circumstances and reactions to this. Data from qualitative inquiry indeed suggests that patients normalise experience by adjusting to HD treatment as they accept being a “patient” [26]. This process may be different for patients in the pre-dialysis phase. Patients at early stages of CKD without KRT are less likely to experience symptoms hence they do not consider themselves as “patient” [50]. However, they tend to perceive CKD as controllable as they can achieve this by complying doctors’ advice, e.g., dietary changes, medication adherence [48]. Also, this may have contributed to increased perceptions of personal control in patients in the pre-dialysis phase. Absence of physical symptoms in the early stages in CKD and belief that disease progression is controllable by healthy lifestyle changes can promote sense of control among patients at early stage of CKD (without KRT) [48,50]. However, this may not relate to perception of timeline (chronicity) necessarily. In line with study by Jansen et al. [20], perception of timeline was predicted to increase in both patient groups at 12 months. Patients in the pre-dialysis phase may have recognised chronic nature of CKD as well as the possibility of commencing dialysis treatment when reduction in kidney function is not well-controlled [48].

Depressive symptom scores among the whole patient group seemed relatively stable over one year period. In line with available research [51], patients receiving dialysis were found to report greater levels of depressive symptoms compared to pre-dialysis patients. Though pre-dialysis may be a time of uncertainty for patients, research has shown several aspects of dialysis that are especially challenging for patients [52,53]. Whilst this is a ‘life line’, any form of dialysis also comes with adjustments in lifestyle and life participation that patients report as problematic. This brings negative outcomes including physical (e.g., fatigue, muscle cramps), emotional and social difficulties (e.g., inability to maintain social activities) [26,54]. This may contribute overall psychological distress.

When selected illness perceptions were evaluated as predictors, only higher levels of perceptions of consequences and emotional response at baseline were found to be related to increased depressive symptoms in patients in the pre-dialysis phase and those receiving dialysis. These findings are supported with similar studies showing association between depressive symptoms and perception of consequences [30,32] as well as emotional response [29] in patients receiving dialysis. Unlike longitudinal findings elsewhere [31] we did not observe significant association between depressive symptoms and perceptions of personal control. It should be noted that patient groups in the current study showed opposite trends of perception of personal control over 12 months when illness perceptions were examined according to phase/treatment (pre-dialysis phase versus dialysis). Having different trends of perceptions of personal control at different stages of CKD may be one of the factors that we did not establish significant association between perception of personal control and depressive symptoms in overall sample. Additionally, unlike longitudinal study by Chilcot et al. [55] we did not find significant association between baseline perception of timeline and increase in depressive symptoms. In their study this was established in patients during their first year on dialysis. However, the current sample included patients already receiving dialysis almost for 5 years (average) and patients in the pre-dialysis phase who already established chronicity of CKD. The overall findings indicate that depressive symptoms are relatively stable overtime in both patients in pre-dialysis phase and patients receiving dialysis. However, this tends to mask differences in trajectories in subgroups of patients related to differences in illness perceptions. There is much similarity between our findings and those of previous studies. There are differences, in particular, in relation to the role of personal control and timeline in influencing trajectory of depressive symptoms. There are multiple potential reasons for this, e.g., differences in dialysis vintage which was higher in the current sample compared to a study in which incident dialysis cohort were examined over one year [31,55]. Also, differences in healthcare settings may play a role, particularly in relation to the availability of psychological support, ongoing patient education and self-management opportunities [25]. These may be particularly relevant Northern Cyprus. Social and cultural differences may also play a role. Although mental health stigma is poorly studied in Northern Cyprus, the limited research indicates that Turkish Cypriot culture is associated with negative attitudes towards mental health issues [56]. This may have played a role in participants’ responses to questions related to depressive symptoms. Nevertheless, it is possible that participants in the current study similar to individuals from collectivist cultures may have different sense-making process of illness in which physical and emotional symptoms are not clearly distinguished [57].

Dialysis symptom burden may vary even over short follow-up periods [58]. Our findings though demonstrated relative stability in patients receiving dialysis over a one year period. HD treatment has been frequently associated with poorer physical functioning due to greater emotional distress, and treatment burden [59]. Our study found similar dialysis symptom burden between patients receiving HD and those receiving PD, perhaps related to the similarity of the groups in terms of age and comorbidity [60]. In support of available evidence [61], a greater number of comorbid conditions at baseline was associated with increase in dialysis symptom burden over time. This is not surprising as comorbidity is seen as one of the most important predictors of clinical outcome among patients with CKD receiving KRT [62]. Complex interactions between multiple conditions likely to cause difficulties in treatment which then may lead to greater symptom severity [63]. Higher level of perception of emotional response at baseline was associated with increased dialysis symptom burden whilst greater perception of personal control was associated with a reduction. This findings are in line with longitudinal study by Covic et al. [24] showing greater perception of personal control and lower level of perception of emotional response at baseline were associated with better physical functioning scores in patients receiving HD. It is known that patients experiencing negative emotional state tend to report greater symptom burden and symptom severity in kidney failure [64]. Similarly, forming greater perception of emotional response to the illness is expected to contribute to poorer illness outcomes [65]. On the other hand, having sense of personal control over illness may provide confirmation of patient role with recognition of responsibilities such as adherence to treatment (e.g., medication, diet) [66]. Perception of having control over some aspects of treatment may have reduced dialysis symptom burden over time in patients receiving dialysis in this sample. On the other hand, we did not observe significant association between baseline perception of consequences and change in dialysis symptom burden over time. This was consistent with two years prospective study by Covic et al. [24] in which perception of consequences was not significant predictor for physical functioning in patients receiving HD. Given average years spent on dialysis (almost 5 years) in this sample, patients may have managed to integrate dialysis into their life. Though dialysis-related symptoms may still be problematic and influence life experience, qualitative research have shown patients accepted that dialysis plays vital role in their survival, which helped to adjust to HD [67].

The findings suggest that illness perceptions offer a framework from which to understand variations in experience of depressive symptoms in patients in the pre-dialysis phase and those receiving dialysis, also symptoms associated with this. Also, data support the utility of the illness perception framework in a specific cultural group and are generally consistent with results of research examining illness perceptions in patients with kidney failure. This is the first study to examine illness perceptions in this patient group within this socio-cultural context. Overall, patients who agreed to participate in this study were willing to engage throughout the research. Compared with the similar studies [20,23,31], baseline response rate was better (78%) despite the sample was research naïve group. Though limited evidence in this cultural context, data suggest that patients are happy to take part in assessment, and so plausible that intervention.

There are some caveats to consider when interpreting the findings. It is known that illness perceptions may vary at different time points. This study had three measurement occasions (i.e., baseline, 6 months, 12 months) hence allowed us to perform linear modelling to observe trajectories of illness perceptions and related experiences such as depressive symptoms and dialysis symptom burden. It should be noted that four or more measurement occasions have been suggested to allow for better understanding with examination of non-linear relationship (e.g., quadratic and cubic growth models) between the variables [68]. Future research may consider this to identify key intervention junctures.

The COVID-19 pandemic may have impacted on this vulnerable patient population, restricting their daily routines [69]. Though patients receiving dialysis had access to care, they were expected to be extra cautious as they regularly visited dialysis unit. Given the greater risk of severe illness due to infection, this patient group may have encountered additional obstacles in managing their overall health, further intensifying the challenges associated with their condition. Patients within the pre-dialysis phase (without KRT) should also be considered as a vulnerable group in this context. Withstanding the added stress that patients may have experienced, the data still point to directions for intervention design based on illness perceptions were also affected during pandemic. Particularly some patients in this group seem to miss their follow-up appointments, this may have contributed to drop–out.

A further factor related to the pandemic that should be noted is the practical implication for the research where follow-up capacity changed from face to face to telephone. Although the data collection mode was changed on the second measurement occasion, patients generally found the telephone better as they could share their experiences in private, not in a hospital setting. Hence the change in data collection mode may have had a positive impact on data collection. Multiple data collection modes have been suggested in longitudinal designs to maintain the response rate [70]. Considering the eligible number of the local patient group at the time of the study (n = 231), and the fact that the sample was a research naïve group, the consent rate at baseline (78%) was high, which was favourable at the third time point (66%). This is critical in terms of representativeness of the sample as well the robustness of study findings which have highlighted the psychological care needs of the patients. The finding that there did not appear to be systematic differences in those who did and did not complete the study suggests that drop out was likely random in nature. The study methods, though responsive to the pandemic need, offer avenues for future research employing similar designs.

This study, for the first time, longitudinally examined illness perceptions of Turkish Cypriot patients with CKD not receiving KRT and those receiving dialysis. Findings indicated that the dialysis group tended to have negative illness perceptions (i.e., greater perceptions of consequences, emotional response and lower personal control) compared to patients in the pre-dialysis phase. Longitudinal data analyses showed that illness perceptions changed over time. However, depressive symptoms and dialysis symptom burden seemed to be relatively stable, though associated with some of the baseline illness perceptions. Drawing on wider illness perceptions research there would be merit in exploring illness perceptions, which are amenable to change as a lever for depressive symptoms and dialysis symptom burden. The findings make the case for the introduction of regular psychological assessment into healthcare provided at the local nephrology departments to help identify negative illness perceptions and emotional distress at early phase. Psycho-educational interventions may be designed to modify negative illness perceptions which respect the socio-cultural characteristics of the patient group.

## Supporting information

S1 Table STROBE Statement—Checklist of items that should be included in reports of cohort studies. (PDF)

S2 Table Descriptive statistics comparing patients who completed the study vs. those who dropped out. (PDF)
