# Supplementary material for: Trajectories of illness perceptions in patients with kidney disease receiving dialysis: Relationship with psychological and physical symptoms
Source: PLoS One. 2025 May 15;20(5):e0323814. doi: 10.1371/journal.pone.0323814 (PMC12080827; doi:10.1371/journal.pone.0323814)
Supplement: S1 Table — (PDF) [file pone.0323814.s001.pdf]

STROBE Statement—Checklist of items that should be included in reports of ***cohort studies***

	Item No	Recommendation	Page no.	Relevant text from manuscript
**Title and abstract**	1	(*a*) Indicate the study's design with a commonly used term in the title or the abstract	2	Abstract
		(*b*) Provide in the abstract an informative and balanced summary of what was done and what was found	2	Abstract
**Introduction**				
Background/rationale	2	Explain the scientific background and rationale for the investigation being reported	3-5	Introduction
Objectives	3	State specific objectives, including any prespecified hypotheses	5	Introduction
**Methods**				
Study design	4	Present key elements of study design early in the paper	6	Methods; Study design and setting
Setting	5	Describe the setting, locations, and relevant dates, including periods of recruitment, exposure, follow-up, and data collection	6-7	Methods; Study design and setting, participants
Participants	6	(*a*) Give the eligibility criteria, and the sources and methods of selection of participants. Describe methods of follow-up	6-7	Methods; Study design and setting, participants
		(*b*) For matched studies, give matching criteria and number of exposed and unexposed	NA	
Variables	7	Clearly define all outcomes, exposures, predictors, potential confounders, and effect modifiers. Give diagnostic criteria, if applicable	9	Methods; Data analysis
Data sources/ measurement	8*	For each variable of interest, give sources of data and details of methods of assessment (measurement). Describe comparability of assessment methods if there is more than one group	7-8	Methods; Data collection
Bias	9	Describe any efforts to address potential sources of bias	NA	
Study size	10	Explain how the study size was arrived at	6-7	Methods; Participants
Quantitative variables	11	Explain how quantitative variables were handled in the analyses. If applicable, describe which groupings were chosen and why	9	Methods; Data analysis
Statistical methods	12	(*a*) Describe all statistical methods, including those used to control for confounding	9	Methods; Data analysis
		(*b*) Describe any methods used to examine subgroups and interactions	NA	
		(*c*) Explain how missing data were addressed	NA	
		(*d*) If applicable, explain how loss to follow-up was addressed	NA	

		(*e*) Describe any sensitivity analyses	NA	
**Results**				
Participants	13*	(a) Report numbers of individuals at each stage of study—eg numbers potentially eligible, examined for eligibility, confirmed eligible, included in the study, completing follow-up, and analysed	11	Results: Participant recruitment and retention, Figure 1
		(b) Give reasons for non-participation at each stage	11	Results: Participant recruitment and retention, Figure 1
		(c) Consider use of a flow diagram	11	Results: Participant recruitment and retention, Figure 1
Descriptive data	14*	(a) Give characteristics of study participants (eg demographic, clinical, social) and information on exposures and potential confounders	11	Results: Baseline patient characteristics
		(b) Indicate number of participants with missing data for each variable of interest	NA	
		(c) Summarise follow-up time (eg, average and total amount)	11	Results; Baseline patient characteristics
Outcome data	15*	Report numbers of outcome events or summary measures over time	13-21	Results: Longitudinal data analyses (Table 2, 3, 4)
Main results	16	(*a*) Give unadjusted estimates and, if applicable, confounder-adjusted estimates and their precision (eg, 95% confidence interval). Make clear which confounders were adjusted for and why they were included	13-21	Results: Longitudinal data analyses (Table 2, 3, 4)
		(*b*) Report category boundaries when continuous variables were categorized	NA	
		(*c*) If relevant, consider translating estimates of relative risk into absolute risk for a meaningful time period	NA	
Other analyses	17	Report other analyses done—eg analyses of subgroups and interactions, and sensitivity analyses	NA	
**Discussion**				
Key results	18	Summarise key results with reference to study objectives	21-26	Discussion
Limitations	19	Discuss limitations of the study, taking into account sources of potential bias or imprecision. Discuss both direction and magnitude of any potential bias	26-27	Discussion
Interpretation	20	Give a cautious overall interpretation of results considering objectives, limitations, multiplicity of analyses, results from similar studies, and other relevant evidence	26-28	Discussion
Generalisability	21	Discuss the generalisability (external validity) of the study results	26-28	Discussion
**Other information**				

Funding	22	Give the source of funding and the role of the funders for the present study and, if applicable, for the original study on which the present article is based	NA
