# Supplementary material for: Trajectories of illness perceptions in patients with kidney disease receiving dialysis: Relationship with psychological and physical symptoms
Source: PLoS One. 2025 May 15;20(5):e0323814. doi: 10.1371/journal.pone.0323814 (PMC12080827; doi:10.1371/journal.pone.0323814)
Supplement: S2 Table — (PDF) [file pone.0323814.s002.pdf]

Table. *Descriptive statistics comparing patients who completed the study vs. those who dropped out.*

Variable	Completed (n = 122)	Dropped out (n = 59)	*t* / χ^2	*p*
Age (mean, SD)	63.1 (14.7)	65.4 (14.2)	0.36	0.337
Gender (male n, %)	83 (45.9)	44 (24.3)	0.81	0.367
Marital status				
Married	92 (50.8)	46 (23.8)		
Single/divorced/widowed	30 (16.6)	16 (8.8)	1.63	0.803
Employment				
Retired	78 (43.1)	41 (22.7)		
Full time/part time employee	10 (5.5)	1 (0.6)	3.24	0.518
Housewife	24 (13.3)	11(6.1)		
Unemployed	10 (5.5)	6 (3.3)		
Comorbidity	2.1 (0.9)	2.1 (0.9)	0.09	0.930
Perception of consequences	6 (3.8)	6.4 (3.9)	0.66	0.505
Perception of timeline	7.7 (2.9)	8.1 (2.7)	0.51	0.607
Perception of personal control	3.5 (2.5)	3.3 (2.5)	-0.45	0.653
Perception of emotional response	4.8 (3.7)	4.9 (3.7)	0.15	0.884
